# Climate change affects the parasitism rate and impairs the regulation of genes related to oxidative stress and ionoregulation of *Colossoma macropomum*

**DOI:** 10.1038/s41598-021-01830-1

**Published:** 2021-11-16

**Authors:** Jaqueline Custodio da Costa, Samara Silva de Souza, Jonatas da Silva Castro, Renan Diego Amanajás, Adalberto Luis Val

**Affiliations:** Laboratory of Ecophysiology and Molecular Evolution (LEEM), National Institute for Research of the Amazon (INPA), Manaus, Amazonas 69067-375 Brazil

**Keywords:** Genetics, Molecular biology, Physiology, Climate sciences

## Abstract

Global climate change represents a critical threat to the environment since it influences organismic interactions, such as the host-parasite systems, mainly in ectotherms including fishes. Rising temperature and CO_2_ are predicted to affect this interaction other and critical physiological processes in fish. Herein, we investigated the effects of different periods of exposure to climate change scenarios and to two degrees of parasitism by monogeneans in the host-parasite interaction, as well as the antioxidant and ionoregulatory responses of tambaqui (*Colossoma macropomum*), an important species in South American fishing and aquaculture. We hypothesized that temperature and CO_2_ changes in combination with parasite infection would interfere with the host’s physiological processes that are related to oxidative stress and ionoregulation. We experimentally exposed *C. macropomum* to low and high levels of parasitism in the current and extreme climate scenarios (4.5 °C and 900 ppm CO_2_ above current levels) for periods of seven and thirty days and we use as analyzed factors; the exposure time, the climate scenario and parasitism level in a 2 × 2 × 2 factorial through a three-way ANOVA as being fish the experimental unit (n = 8). An analysis of gill enzymatic and gene expression profile was performed to assess physiological (SOD, GPx and Na^+^/K^+^-ATPase enzymes) and molecular (*Nrf2, SOD1, HIF-1α* and *NKA* α1a genes) responses. A clear difference in the parasitism levels of individuals exposed to the extreme climate scenario was observed with a rapid and aggressive increase that was higher after 7 days of exposure though showed a decrease after 30 days. The combination of exposure to the extreme climate change scenario and parasitism caused oxidative stress and osmoregulatory disturbance, which was observed through the analysis of gene expression (*Nrf2, SOD1, HIF-1α* and *NKA* α1a) and antioxidant and ionoregulatory enzymes (SOD, GPx and Na^+^/K^+^-ATPase) on the host, possibly linked to inflammatory processes caused by the high degree of parasitism. In the coming years, these conditions may result in losses of performance for this species, and as such will represent ecological damage and economical losses, and result in a possible vulnerability in relation to food security.

## Introduction

Global warming represents one of the greatest threats to ecosystems since it affects biodiversity at all levels^1^. Climate projections forecast an increase in global mean temperatures of around 6 °C by the end of this century. This situation will clearly affect the freshwater environment^2^, particularly the fish due to the negative effects on physiology, survival, immunity and greater disease susceptibility^3,4^.

Infectious diseases cause significant population declines in wild and captive animals^5^. Small flatworms, such as the Monogenea, are one of the main causes of disease outbreaks and economic losses^6^. These parasites have a direct, single-host life cycle, and are thus able to multiply rapidly in aquaculture environments^7^. Most monogeneans live freely on the fish’s skin and feed on mucus, epithelial cells, and gills resulting in damage to the host tissue such as deep wounds in the epidermis, basement membrane and into the dermis^7,8^.

Diseases in highly commercialized species cause significant economic losses every year, especially in the fish farming sector where high densities of stressed animals and poor water quality provides ideal conditions for outbreaks of disease, including those caused by monogeneans^5,9^. For this reason, in recent years, the topics of climate change and infectious diseases in aquatic animals have gained special attention, but the evidence regarding the connection is still scarce^10–12^. Some studies have shown that in fish climate change aggravates infectious diseases caused by bacteria^13,14^ and parasites^15–17^. However, information is lacking when it comes to Neotropical fish, especially those from the Amazon.

The oxidative stress appears to play a major role in the pathogenesis and progression of many infectious diseases^18^. In our preliminary study^16^, we observed that the degree of parasitism by Monogenea in tambaqui (*Colossoma macropomum*), a highly commercialized species in the Amazon region, is differentially affected by the climate change scenario; fish kept in the extreme climate scenario show a significant increase in the level of parasitism, which is accompanied by an inflammatory response, as well as by overexpression of pro-inflammatory cytokines such as *IL-1β* and negative regulation of anti-inflammatory cytokine such as *IL-10*. Taken together, the data suggest that the activation of inflammatory mechanisms seems to be an adaptive response of the species to try to deal with the increase in parasitism imposed by climate change, which evidences a key role of the genes that regulate inflammation.

Inflammation and oxidative stress are inextricably linked. Oxidative stress plays a key role during inflammatory reactions and is considered an important pathophysiological process promoted by reactive oxygen species (ROS) production^19,20^. Enzymes and genes related to the antioxidant system play a fundamental role as the main defensive line in the response to oxidative stress by eliminating the ROS and in the maintenance of homeostasis^21,22^. The antioxidant capacity is partly regulated by a wide variety of transcription factors, including NF-E2-related factor 2 (Nrf2), which modulates the transcription of type II detoxifying enzyme genes, such as *SOD1*, which encodes Cu/Zn superoxide dismutase, and is a crucial combatant to augmented oxidative stress and prevents the free radical exchange reaction of relatively toxic superoxide by accelerating its oxidation to non-toxic hydrogen peroxide^23,24^.

ROS and antioxidant mechanisms also play an important role in regulating *HIF-1*α (hypoxia inducible factor)^20^. HIF is a key regulator that plays a central role in O_2_ homeostasis, and is able to transcriptionally control the expression of more than 1000 genes that are involved by binding to DNA sequences that contain hypoxic response elements (HRES)^25^. *HIF-1α* can be activated/stabilized in response to increased ROS levels and is also induced by the parasite damage to the gills^26^. It is particularly important when looking at the relationship between *HIF-1α* and genes related to inflammatory mechanisms. Cytokines can activate *HIF-1α* expression in a ROS-dependent manner^27^. For fish, the role of *HIF-1α* on inflammation is still unclear and may vary between different species, both promoting and inhibiting inflammation^28,29^.

Recent studies have reported not only the negative effects of parasitism on the antioxidant system, but also its negative effects on fish ionoregulation^30–32^. These results deserve to be highlighted, mainly when it comes to infection by monogeneans, since they promote severe damage to the gills of the hosts, which is the main organ related to ionoregulatory processes in fish^7,8^. Gills are a physical barrier that largely depend on the structural integrity of epithelial cells and intercellular tight junctions. The structural integrity of epithelial cells is related to antioxidant ability, gas exchange, ion regulation, and acid–base balance, and the damage caused by the parasite can compromise all of these functions^33,34^.

Na^+^/K^+^-ATPase (NKA) is essential to ensure efficient ion transport in osmoregulatory epithelia in order to maintain ionic homeostasis. In the osmoregulatory tissues of aquatic organisms, the electrochemical gradient generated by the NKA provides a driving energy for many transport systems via channels and transporters that are localized in the apical and basolateral membranes^33,35^. The *NKA α-1a* subunit is expressed in lamellar mitochondrion-rich cells in the gills and has been reported as the gill-specific gene involved in ion absorption in freshwater fish^36^. Some mammalian studies report that inhibition of *NKA α-1a* production increases oxidative stress and intracellular ROS formation^37^. However, there is no data regarding the role of NKA in the face of exposure to climate change scenarios and parasites that cause severe branchial tissue damage, one of the most responsive organs to ionoregulation. Monogenea may interfere with all of these crucial molecular/physiological functions in the host, as mentioned above.

Tambaqui (*Colossoma macropomum*) is the main native species farmed in South American continental waters^38^. Similar to several fish species cultivated worldwide, tambaqui are highly affected by monogenean infections and thus constitutes a good biological model for evaluating environment-parasite-host interaction. We previously demonstrated that climate change will favor the proliferation of monogeneans, especially in less-parasitized tambaqui, and promotes the activation of inflammatory mechanisms^16^. These results motivated us to perform this second study.

Thus, herein, we investigated whether different periods of exposure to climate change scenarios (7 and 30 days) and two degrees of parasitism (low and high) would affect the environment-parasite-host interaction and the tambaqui antioxidant and ionoregulatory responses. We hypothesized that (i) an increased rate of parasitism caused by the extreme climate scenario would be maintained during the 30 days of exposure and (ii) the inflammation caused by the high parasitism would affect the expression of genes and enzymes related to antioxidant and ionoregulatory responses.

## Results

### Parasitological analysis

Two degrees of parasitism were considered after the parasitological analysis: low, 1–32 monogeneans per fish, and high, over 32 monogeneans per fish. The species identified in this study were *Mymarothecium boegeri*^39^ and *Notozothecium janauachensis*^40^.

**Figure 1:**
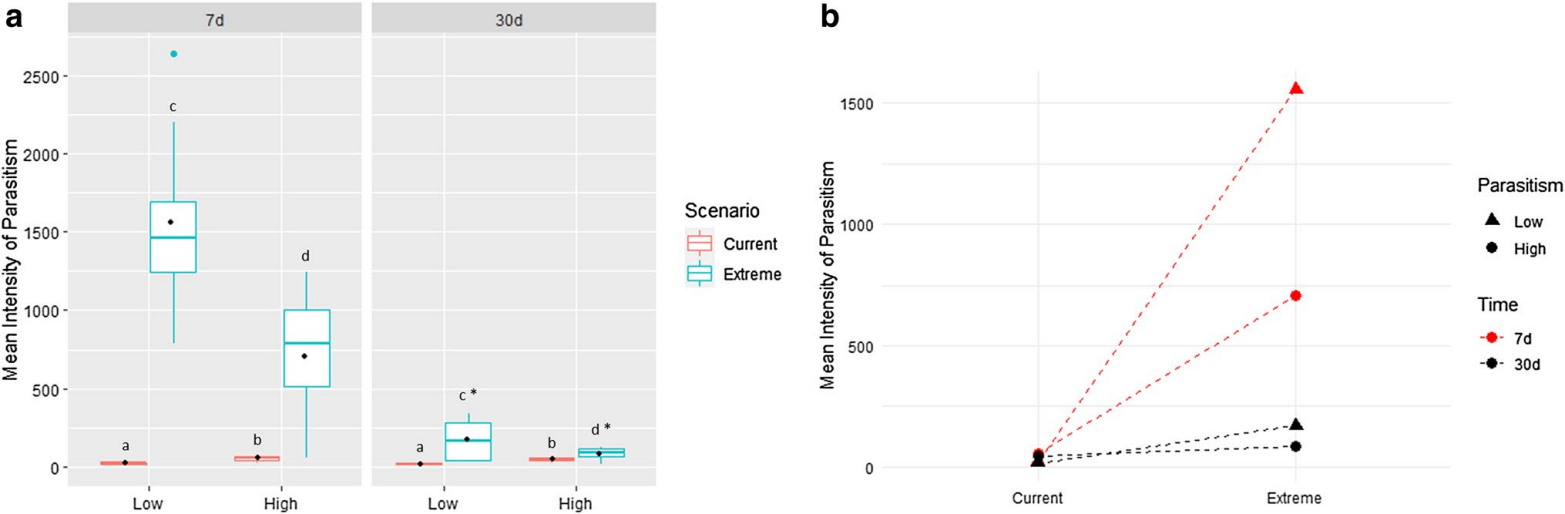
Mean intensity of parasitism in fish exposed to 7 and 30 days of extreme climate scenario (**a**) and a plot indicating the interaction between the parasitism levels and the period of exposure (**b**). Low (LG) and High (HG) indicate parasitism level. Boxes with different letters indicate differences between scenarios and asterisks indicate differences between exposure periods (*p* < 0.05). A black dot in the box indicates the average of each group.

The parasitological analysis revealed that the mean intensity of parasitism was significantly increased in both groups exposed to the extreme scenario in 7 and 30 days (*p* < 0.001; F = 175.804) with interaction between the scenarios and parasitism (*p* < 0.001; F = 28.993). However, after 30 days there was a decrease, which can be seen in Fig. 1. The low-parasitized animals subjected to the extreme scenario presented a greater increase in the mean intensity of parasitism as was observed in our previous study^16^ and this difference was maintained in 30 days.

### Antioxidant enzymes

**Figure 2:**
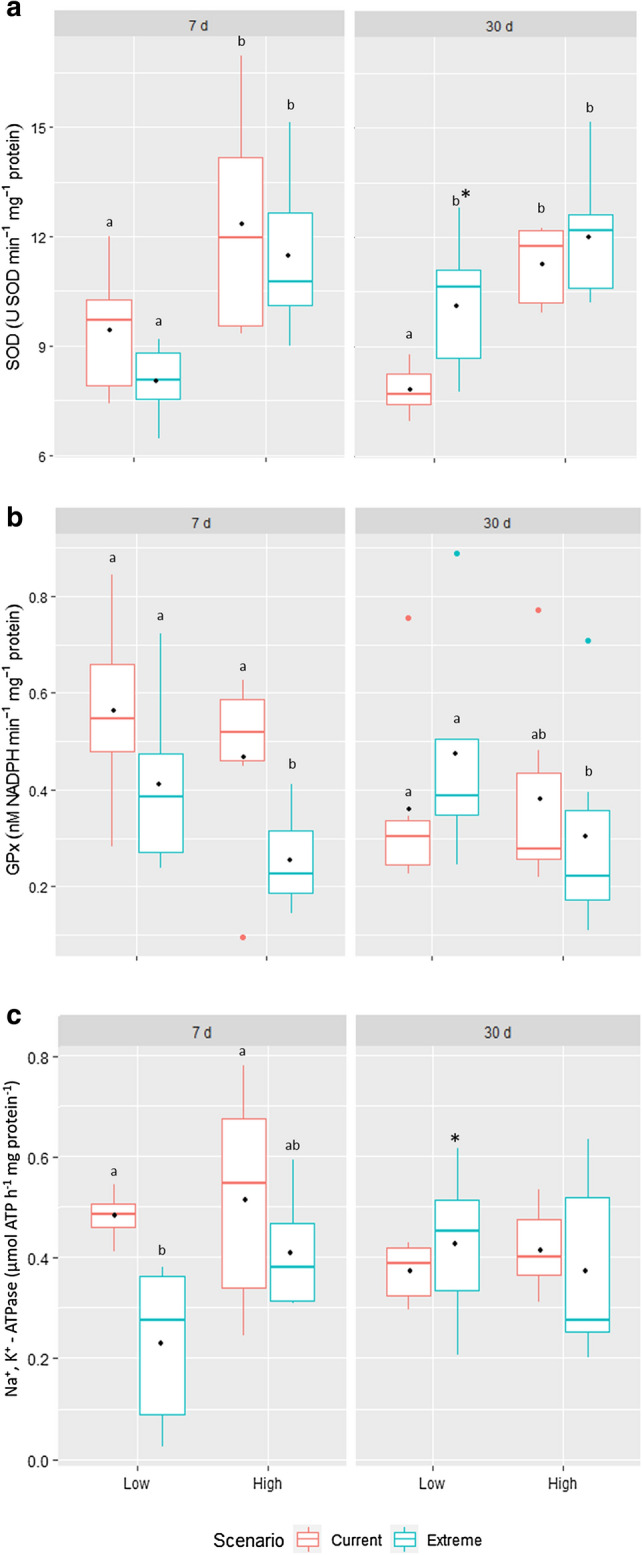
Enzymatic activity of SOD (**a**), GPx (**b**) and NKA (**c**). Low (LG) and High (HG) indicate parasitism level. Boxes with different letters indicate differences between scenarios and asterisks indicate differences between exposure periods (*p* < 0.05). A black dot in the box indicates the average of each group.

An increase of SOD enzyme in activity was observed only in fish in the high parasitism group (HG) in the current scenario when compared with the low parasitism group (LG) after 7 days (*p* = 0.020; F = 5.863). After 30 days, the activity remained constant and showed an increase in the LG (*p* = 0.006; F = 8.536) (Fig. 2a). There was no interaction of factors (*p* = 0.719; F = 0.131). For GPx (Fig. 2b), after 7 days, there was a decrease in activity in the HG in the extreme scenario compared to the current scenario (*p* = 0.030; F = 5.065) and the LG in the extreme scenario (*p* = 0.048; F = 3.258). The same was observed for the HG after 30 days in the extreme scenario when compared with the LG (*p* = 0.028, F = 5.849). There was no interaction of factors (*p* = 0.577; F = 0.316). The NKA activity was lower in the LG after 7 days (*p* = 0.034; F = 3.095), however it showed an increase in the group after 30 days (*p* = 0.005; F = 4.159) and interaction was observed between climate scenarios and parasitism (*p* = 0.014; F = 6.536) (Fig. 2c).

### Gene expression analysis

**Figure 3:**
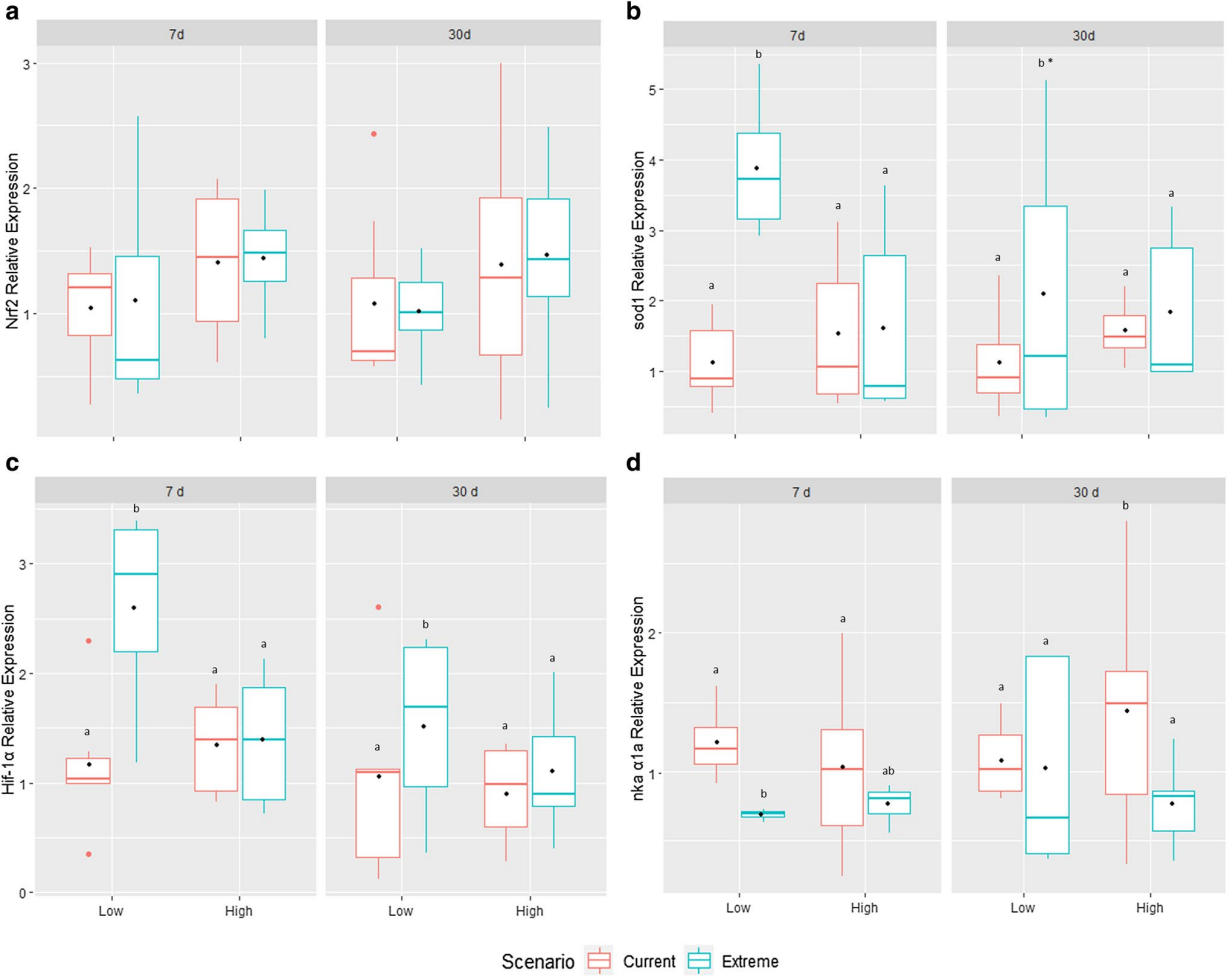
Gene expression of *NRF2* (**a**), *SOD1* (**b**), *HIF-1α* (**c**) and *NKA α1a* (**d**) in the gills of *Colossoma macropomum* exposed to the extreme climate scenario as foreseen by IPCC (2014), compared to current scenario. Boxes with different letters indicate differences between scenarios and asterisks indicate differences between exposure periods (*p* < 0.05). Low (LG) and High (HG) indicate the degree of parasitism after seven and thirty days of exposure to the experimental conditions. A black dot in the box indicates the average of each group.

There was no difference in expression in any of the conditions tested for *Nrf2* gene (Fig. 3a). For *SOD1* after 7 days, the expression increased in fish from the LG maintained in the extreme scenario (*p* = 0.014; F = 6.590) when compared with the same group maintained in the current one (Fig. 3b). After 30 days, the increase in the LG remained constant in fish subjected to the extreme scenario (*p* = 0.053; F = 3.984). The greatest influence was caused by the degree of parasitism, followed by the scenario. There was no interaction of factors (*p* = 0.147; F = 2.186). *HIF-1α* showed an increase in expression also in the LG of the extreme scenario after 7 days (*p* = 0.034; F = 4.934), and a downregulation in the same group after 30 days (*p* = 0.054; F = 4.023; no interaction of factors: *p* = 0.193; F = 1.772), but the difference was maintained between treatments, displaying the same expression pattern as *SOD1* (Fig. 3c). A decrease in the expression of *NKA α1a* in the LG after 7 days of exposure to the extreme scenario (Fig. 3d) was observed (*p* < 0.001; F = 19.057) but, in contrast, in the same group, after 30 days, there was an upregulation (*p* < 0.001; F = 21.036). There was also downregulation of the HG group after 30 days of exposure to the extreme scenario concerning the same group in the current scenario and interaction was observed among exposure period and scenario (*p* = 0.029; F = 5.253) and between the scenario and parasitism (*p* = 0.018; F = 6.237).

**Figure 4:**
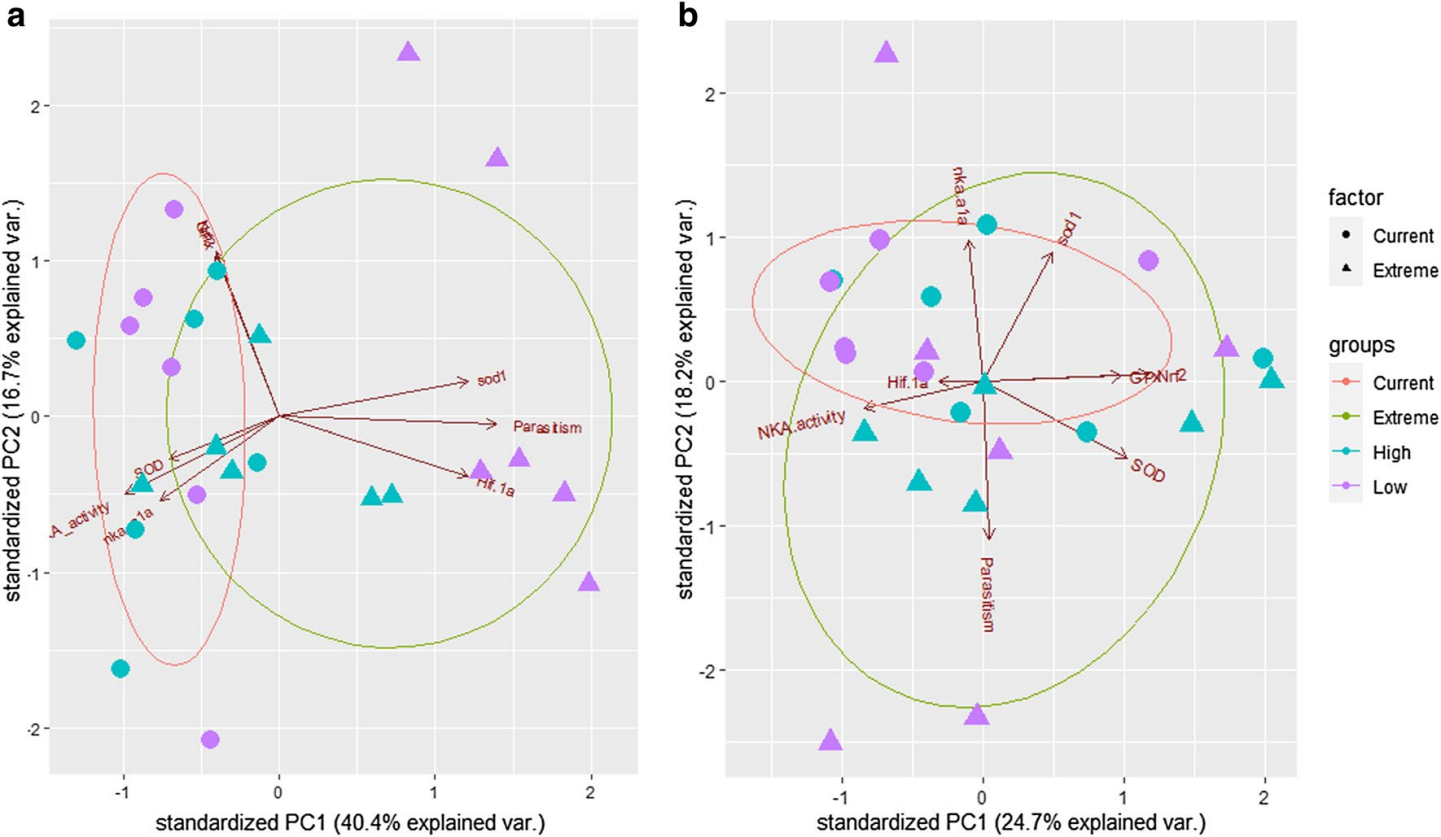
Principal components analysis (PCA) for juvenile tambaqui *Colossoma macropomum* considering distribution patterns between scenario and parasitism rate (Low (LG) and High (HG)) after seven (**a**) and thirty days (**b**) of exposure.

The principal components analysis (Fig. 4) demonstrated that gene expression patterns are different between climate scenarios and also between different parasitism intensities after 7 days. The extreme climate scenario, parasitism, and gene expression (*SOD1* and *HIF-1α*) are further related by PC1 (40.4%) after 7 days. After 30 days, we observed that there is a change in the observed patterns. While climate scenario did not show much difference, it is still possible to observe that parasitism and SOD correspond to the extreme scenario and are related by PC1 (24.7%) and NKA activity to PC2 (18.2%). The cumulative explained variance of PC1 and PC2 was 57.1% after 7 days and 42.9% after 30 days. Therefore, these findings corroborate the results from the ANOVA data.

## Discussion

**Figure 5:**
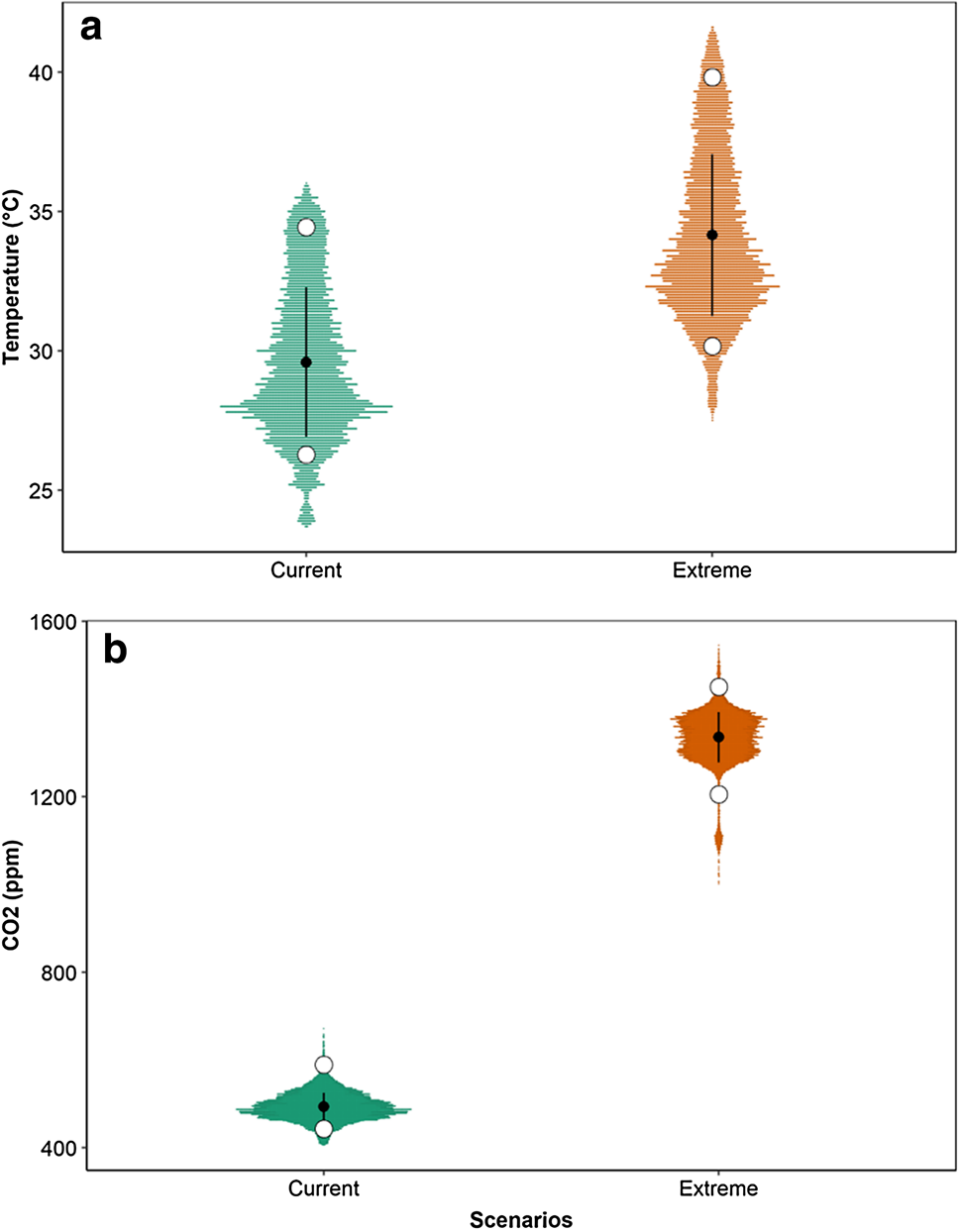
Variations in air temperature (**a**) and CO_2_ concentration (**b**) in the two environmental rooms, as proposed by IPCC (2014) for the year 2100 over the 30-day experimental period.

Several environmental stressors, mainly those caused by climatic factors, such as temperature and CO_2_, affect various molecular and physiological processes in fish^41–43^. Our findings are in agreement with our preliminary results^16^, which indicate that the exposure of tambaqui to the extreme climate scenario for 7 days induced a significant increase in the parasitic load of both groups (high and low parasitism level) with emphasis on the low group, and the same was observed in the present study. However, after 30 days, we observed a decrease in the parasitic load in both groups, but this was even greater when compared to the current scenario (see Fig. 5). This can potentially be explained by chronic thermal stress on the parasite, which can be a predominant factor that harms its survival by causing damage to its physiological functions, as observed in *Poecilia reticulata*^44^, when infected by the monogenean *Gyrodactylus turnbulli,* there was a decrease in parasitic load when exposed to a temperature of 32 ºC, which for this fish is considered extreme. Brazenor and Hutson^45^ also observed that in warmer waters (31 ºC) *Neobenedenia girellae* parasitizing *Lates calcarifer* had its life cycle reduced and thus, led to fewer stages of transmission. Thus, after 30 days of exposure to the extreme climatic scenario, the decrease in the parasitic load observed here may be the result of direct effects of climatic factors on the parasite and not an adaptive response of the fish.

We also observed that the tambaqui were susceptible to oxidative damage when exposed to the extreme climate scenario plus parasitism. Fish from the LG exhibit a marked increase in *SOD1* mRNA levels, especially when submitted to the extreme scenario for 7 days, though no effect was observed on *Nrf2* expression (Fig. 3a, b). Our findings suggest that parasitism and the climate change scenario induce oxidative damage that influences the increase in S*OD1* mRNA levels. Sun et al.^21^ demonstrated that thermal stress induced both oxidative damage and positive regulation of antioxidant genes in *Micropterus salmoides*, including *SOD1*. Oxidative stress resulting from exposure of fish to parasites has also been reported^30,46,47^, but the effects of parasitism on the expression of genes related to antioxidant processes are not clear.

The literature reports increased expression of antioxidant genes through the Nrf2-ARE pathway^48^; however, in our study *SOD1* expression increases, but it is not accompanied by differential transcriptional activation of *Nrf2*. Other studies have also failed to observe a clear relationship between the levels of *Nrf2* and *SOD1* mRNA in fish^18,49^. Mukaigasa et al.^49^ suggest that SOD may not be a direct target for *Nrf2* since, when evaluating the expression of the gene in zebrafish with a mutation in *Nrf2*, they observed an expression similar to that described in wild fish.

Another possible explanation for the maintenance of transcriptional levels of *Nrf2*, similar to that which occurs in non-stressed groups, is the fact that this gene not only executes via oxidative stress prevention, but also has an anti-inflammatory function (reviwed by Ma^50^). In mammals, *Nrf2* has been shown to inhibit inflammation by blocking NF-κB and consequent inhibition of pro-inflammatory cytokines (reviewed by Hennig et al.^51^; Ma^50^). In our previous study, we observed that the exposure of parasitized tambaqui to climate change scenarios for 7 days resulted in increased expression of *IL-1β* and *HSP70*, pro-inflammatory genes, as well as decreased expression of *IL-10*, anti-inflammatory cytokine, suggesting the activation of inflammation^16^. As inflammatory processes are associated with the production of reactive oxygen species (ROS)^51^, it is expected that positive differential activation of *Nrf2* triggers an antioxidant response, and limits the negative effects of ROS, which would also limit the formation of the inflammasome by blocking the expression of pro-inflammatory genes. We propose that the maintenance of similar transcriptional levels of *Nrf2* between the groups is an attempt to favor activation of the inflammatory process in detriment of the antioxidant response.

This suggestion is supported when we evaluate the activity of antioxidant enzymes. The SOD activity increases after 7 days in the HG in both scenarios, the same being observed for 30 days. However, there was an increase in the LG after 30 days (Fig. 2a) in the extreme scenario, while the GPx activity shows a decrease in highly parasitized fish after 7 and 30 days under the extreme scenario, and remains similar in the other groups (Fig. 2b). The influence of temperature and parasitism on antioxidant responses and oxidative status can vary depending on the tissue, period of exposure, level of parasitism and temperature increase^30,52,53^. In the species *Seriola lalandi*, infection by *Neobenedenia melleni* did not promote an increase in SOD activity^53^. Similar results were observed in the gills of *Siganus oramin* parasitized by *Cryptocaryon irritan*s^54^. However, in the species *Pangasianodon hypophthalamus,* when parasitized by *Thaparocleidus* sp., there was an induction of oxidative stress biomarkers, including SOD activity^30^, which is similar to that observed in our study. Klein et al.^52^ reported the absence of changes in SOD activity and a reduction in branchial GPx activity in *Notothenia coriiceps* that were exposed to temperature increases, and the authors also suggest that fish are unable to positively regulate the activity of antioxidant enzymes in gills under thermal stress conditions.

In the species *Pomatoschistus micros*, the combination of parasitism and an increase in temperature also did not affect SOD activity^46^. Knowing that both parasitism and increased temperature result in oxidative stress^21,46,47^, the results observed in our study indicate an attempt by the fish to eliminate ROS, which is evidenced by the increase in the SOD activity since it is the first line of enzymatic defense against oxidative stress, and whose activity results in the dismutation of the superoxide anion in H_2_O_2_ and H_2_O. This was not observed for GPx, which is the peroxidase involved in the degradation of H_2_O_2_ and other peroxides^55^. The changes observed in the activities of antioxidant enzymes are an indication that parasitism and climate change promoted an increase in the production of ROS and oxidative stress in tambaqui, which, in turn, can guarantee greater efficiency in the inflammatory process triggered by the combination of parasitism and climate change, as evidenced by Costa and Val^16^.

In addition, the data also shows a clear lack of integration between levels of transcripts and enzymatic catalytic activity, mainly after 30 days of exposure. This inverse pattern between mRNA levels and enzyme activity for SOD was observed in fish exposed to different environmental stressors^56–58^. Liu et al.^56^ demonstrated that for zebrafish, the increase in transcripts of antioxidant genes was accompanied by inhibition in the activity of the respective enzyme antioxidants. Similar results have been reported for the species *Ctenopharyngodon idella*^58^. Regoli et al.^57^ suggested that the mRNA levels of antioxidant enzymes may represent a specific cellular response at a given time, but not the outcome of the response to a stressful condition. Contradicting responses in mRNA levels and antioxidant enzyme activity may be related to the transcription’s vulnerability to variation in cellular conditions imposed by metabolite status, exposure period, type of stress and even variations inherent to the species, especially because the transcription is transitory, while enzymatic catalysis is more durable and subject to additional regulatory processes^56^. Therefore, the inconsistency between enzyme activity and levels of transcripts shows the complexity of responses to oxidative stress, and these responses may not be associated with transcriptional variations, but with post-translational changes^57^.

The upregulation of *HIF-1α* mRNA in the LG of the extreme scenario after 7 and 30 days was possibly due to the presence of factors such as ROS and inflammatory cytokines, and this can be supported by the result we obtained with the overexpression of *SOD1* in the same groups (Figs. 3b, c, 4a). Increases in *HIF-1α* transcripts occurred only in the LG whose level of parasitism increased in the extreme scenario, which is greater than that observed in the HG in the same scenario (Fig. 1a), for which the levels of *HIF-1α* mRNA are also lower (Fig. 3c). The transcriptional behavior of *HIF-1α* observed here is the same as that observed for the pro-inflammatory genes (*HSP70* and *IL-1β*), and contrasts with the expression of anti-inflammatory genes (*il-10*) observed in our previous study after 7 days^16^. This suggests that the positive regulation of *HIF-1α* in the LG exposed to extreme scenario may be the result of the release of pro-inflammatory cytokines due to the parasitic load that increased considerably in these fish, contrary to what was initially observed in the HG. According to Zepeda et al.^27^, even if there is no hypoxia, *HIF-1α* activation can occur through cytokines in ROS-dependent mechanisms. A pattern similar to that observed after 7 days occurred after 30 days also in the extreme scenario with the expression of *HIF-1α,* suggesting that the inflammatory response can continue to be active in chronic conditions. In zebrafish, Ogryzko et al.^29^ suggest that the stabilization of *HIF-1α* promotes an increase in *IL-1β* transcription and contributes to host protection, since it is part of an immediate pro-inflammatory response. However, as described by Guan et al.^28^ for *Boleophthalmus pectinirostris*, activation of *HIF*-*1α* and pro and anti-inflammatory cytokines may vary between different species and, in this species, increased *HIF-1α* expression suppressed *IL-1β* expression and upregulated *IL-10*. We propose that the increased degree of parasitism caused by exposure to the extreme climate scenario led to oxidative stress as well as increased inflammatory cytokine levels, thus, increasing the expression of *HIF-1α*. For fish, few studies show a clear relationship among *HIF-1α*, oxidative stress, and infectious diseases. Piazzon et al.^59^ showed that *Sparicotyle chrysophrii* (Monogenea) parasitizing *Sparus aurata* induced hypoxia in the host, which caused the expression of oxidative stress genes, and is associated with upregulation of *HIF-1α*. *Haliotis fulgens*, on the other hand, exposed to hypercapnia, hypoxia and a temperature of 32 °C displayed an upregulation of *HIF-1α* as well as antioxidant genes such as *SOD*^60^. Our results are in agreement with Wang et al.^61^ who observed a relationship between the regulation of *HIF-1α* and defense against infection by the bacterial pathogen *Streptococcus agalactiae* in tilapia in the perspective of global warming.

Unlike *HIF-1α* and *SOD1*, *NKA α1a* showed downregulation in the LG when exposed to the extreme scenario for 7 days; however, after 30 days, the expression returns to normal levels when compared to the current scenario, with the exception of the HG group that remained downregulated (Fig. 3d). As an ion transport pump, NKA is not only crucial for maintenance of the ionic homeostasis, but also plays a critical role in cellular function and signaling^62^. Thus, the downregulation observed here could be a consequence of the damage that the parasite causes to the branchial tissue and this may have affected the normal functioning of mitochondrion-rich cells that then lost their ability to induce *NKA* α1a expression as a result of disruption and failure in molecular machinery. We also speculate that all this downregulation could also be interpreted as a shutdown of the metabolism, as part of a hypometabolic response to concentrate the energy on the immune response. For human cells, the downregulation of *NKA* α1a is associated with increased oxidative stress^37^. Consequently, as already mentioned above, we observed an increase in *SOD1* in the extreme scenario, which is indicative of oxidative stress. Few studies have analyzed the expression of the *NKA α1a* gene in fish and the majority have studied it only in relation to the effect of exposure to salinity. However, the results of Tomalty et al.^64^ differ from our results, since they observed that this gene was upregulated in *Oncorhynchus tshawytscha* under heat stress of 25 °C. *NKA α1a* mRNA was also differentially expressed in blue rockfish (*Sebastes mystinus*) exposed to high *p*CO_2_ and hypoxia, which suggests that the fish effectively use compensatory mechanisms to contend with climate change stressors^65^. This is the first study to analyze the expression of the *NKA* α1a gene in parasitized fish and exposed to synergistic effect of the forecasted 2100 climate scenario (increased temperature and CO_2_) and parasitism.

As well as the results of gene expression, we found a decreased activity of Na^+^/K^+^-ATPase in the branchial tissue in the LG after 7 days also in the extreme scenario, but in the same group after 30 days this had increased. This reduction in activity of Na^+^/K^+^-ATPase after 7 days can be explained in the same way as for gene expression. Severe tissue damage caused by the high parasitism rate driven by the climate change scenario led to an inflammatory stage that impaired the functioning of mitochondrion-rich cells. Kumar et al.^66^ reported that the Pangasius catfish infected by Monogenea reduced NKA activity and they associated this reduction with cellular stress and degradation by toxins released by the parasite itself, which impairs the synthesis. *Sparus aurata* exposed to an outbreak of Amyloodiniosis also showed reduced NKA activity, due to anoxia induced by parasitism that led to ionoregulatory failure^67^. The increase that we observed after 30 days in the LG in the extreme scenario may be a response to the reduced parasitism rate, which lead to a return to normal activity levels.

Evidently, acclimation of the fish to a scenario of progressive changes in temperature and CO_2_ concentrations that simulates a real-life scenario may result in adjustments of responses, providing effective acclimatization to the transitioning environment, and if these predictions are confirmed, our results support the occurrence of the effects observed in animals.

In summary, this study reports the effects caused by the forecasted 2100 climate scenario (increased temperature and CO_2_) after seven and thirty days of exposure associated with two levels of gill parasitism by monogeneans in tambaqui. The present study suggests that climate changes cause a rapid increase in parasitism during seven days, which decreases after thirty days, but remains higher than that of the control group. We also show clear evidence of regulation of genes associated with oxidative stress and inflammation, physiological stress, and ionoregulatory problems associated with a rapid and aggressive increase in parasitic infection caused by the climate change scenario. However, gene expression should not be confused with the proteins that execute the processes.

## Material and methods

### Ethics statement

The experimental procedures were approved by the Ethics and Animal Welfare Committee (CEUA) of the Brazilian National Institute for Research of the Amazon (INPA), Manaus, AM, Brazil, under protocol number 053/2017 and were conducted in accordance with all relevant guidelines and regulations applicable. The study also followed the recommendations in the ARRIVE guidelines^68^.

### Fish acquisition and acclimation

A total of 64 juvenile tambaqui (weight: 45.25 ± 3.43 g and length: 14.19 ± 1.15 cm) were obtained from a local fish farm (Fazenda Santo Antônio: 02° 44′ 802″ S; 059° 28′ 836″ W, Amazonas, Brazil) and transferred to the Laboratory of Ecophysiology and Molecular Evolution (LEEM) at INPA. Fish were acclimated for at least 3 weeks before the experiment in 310 L tanks with continuous aeration and water flow, the temperature of 26.2 ± 0.8, dissolved oxygen 6.5 ± 0.5 pH 6 ± 0.5 in order to recover from transport stress. Fish were fed once a day with a commercial diet (2-4 mm pellets, 36% of crude protein) during this period.

### Experimental design

These fish were naturally parasitized and after the acclimation period were treated with 3 g L^−1^ of salt for 15 min during three consecutive days according to Schelkle et al.^69^ to decrease the animals’ parasitic burden and thus establish two degrees of parasitism according to the protocol used by Costa and Val^16^.

After defining the two degrees of parasitism (low and high group, which we abbreviate to LG and HG, respectively), the animals were transferred to two real-time controlled environmental rooms as described by Costa and Val^16^. Each room simulated the current (current temperature and CO_2_ levels) and extreme (RCP8.5) scenarios according to the Fifth IPCC Assessment Report for the year 2100^2^. The current conditions simulate the same conditions occurring in a forested area of the Amazon without human influence, with data acquisition by Fieldlogger 512k (Novus Produtos eletrônicos LTDA) every two minutes. The extreme climate room was set to 4.5 °C and 900 pmm CO_2_ above the current conditions (Fig. 5). The artificial light–dark cycle was 12:12, and humidity was set as a derived condition. Prior to the experiment, the gills, skin, and fins were carefully scraped with a coverslip and observed under an optical microscope, and underlying subclinical infection and/or the presence of other parasites were assessed.

The juveniles were distributed in eight 60 L PVC tanks in four replicates per treatment (low and high levels of parasitism) containing four individuals each tank, in both scenarios. The fish were exposed to each climate room for seven and thirty days in October 2018 (Amazon dry season). After each exposure period, two fish were removed from each tank, with a total of eight fish per scenario and treatment being collected (n = 8). The volume of water was adjusted after collecting the fish. The illustration in supplementary information (Fig. S1) demonstrates the experimental setup performed in this study. Ammonia accumulation was avoided by partial water renewal throughout the experiment. The pH, O_2_ and CO_2_ levels and temperature of the water were measured daily (Table 1). All animals were fed once a day with a commercial diet with 36% crude protein during the experiment. Subsequently, the fish were anaesthetized, weighed, measured and euthanized by rapidly severing their spinal cord with a scalpel for tissue sampling. Gill samples were collected using sterile tweezers and scissors, and one side of each fish was immediately immersed in liquid N_2_ and stored in an ultra-freezer at − 80 °C for biochemical and genetic analysis.

**Table 1:** Temperature, O_2_, CO_2_, and pH of the water in the aquariums in the experimental climate rooms.

Scenario	Water			
	Temperature (°C)	O_2_ (mg L^−1^)	CO_2_ (ppm)	pH
Current	26.1 ± 1.3	6.28 ± 1.0	9.55 ± 0.9	6.1 ± 0.2
Extreme	30.3 ± 0.7*	5.45 ± 0.8*	14.83 ± 1.2*	5.6 ± 0.5

The two environmental rooms were computer-controlled in real-time to simulate current environmental conditions and the extreme climate scenario (RCP8.5, plus 4.5 °C and 900 ppm CO_2_) as proposed by IPCC (2014) for the year 2100.

*indicates significant difference from current scenario (Student’s t-test, *p* > 0.05).

### Parasitological analysis

The branchial arches from one side of each fish were sampled, separated and placed individually in plastic vials of 60 mL containing formalin (1:4000). The parasites were scraped from the gills and the contents of the plastic vials were counted under a stereomicroscope in a Petri dish. After counting, the parasites were stored in microtubes in 70% alcohol for later identification according to Rawson and Rogers^70^. To estimate the total amount of parasites per fish, the result was doubled. The prevalence and mean intensity of parasites were calculated according Bush et al.^71^.

### Biochemical analysis

#### Homogenate preparation

To determine superoxide dismutase (SOD) and glutathione peroxidase (GPx) activity, gill samples were homogenized in a cold buffer containing (in mM): 200 Tris-Base, 1 EDTA, 1 dithiothreitol, 500 sucrose, 150 KCl, pH 7.6) and then centrifuged at 9000*g* for 30 min at 4 °C.

#### Antioxidant enzymes

The glutathione peroxidase (GPx) activity was determined based on the oxidation of NADPH in the presence of GSH (0.95 mM) and H_2_O_2_ at 340 nm, according to the method described by Hopkins and Tudhope^72^ and activity was calculated as nmol of oxidized NADPH min^−1^ mg protein^−1^ using the molar extinction coefficient of 6.22 mM^−1^ cm^−1^.

Superoxide dismutase (SOD) activity was quantified based on the inhibition of the cytochrome c reduction rate by the superoxide radical at 550 nm and 25 °C, according to the method described by Turrens^73^. Enzyme activity is expressed as U SOD mg protein^−1^, where 1 U of SOD corresponds to the quantity of enzyme that promoted the inhibition of 50% of cytochrome c.

#### Gill Na^+^/K^+^-ATPase activity

The activity of this enzyme was determined by NADH oxidation in an enzymatic reaction coupled to the hydrolysis of ATP^74^. The assay is based on the inhibition of NKA activity by ouabain (2 mM). Gills were homogenized (1:10 w/v) in buffer (pH 7.5) containing (in mM): sucrose 150, imidazole 50, EDTA 10 and deoxycholic acid 2.5, and centrifuged at 2,000 × g for 7 min at 4 °C. Supernatants were added to a reaction mixture containing (in mM): imidazole 30, NaCl 45, KCl 15, MgCl2 3.0, KCN 0.4, ATP 1.0, NADH 0.2, fructose-1,6-bisphosphate 0.1, PEP 2.0, with 3 U mL^−1^ pyruvate kinase and 2 U mL^−1^ lactate dehydrogenase. Samples were run with and without ouabain. Absorbance was read over 10 min at 340 nm in a spectrophotometer (Spectra max Plus, model 384, Molecular Devices®, USA). Na^+^/K^+^-ATPase activity was calculated by the differences between total activity and activities with the ouabain inhibitor.

#### Total proteins

The total protein in gill samples was quantified in a spectrophotometer at 595 nm (Spectra max Plus, model 384, Molecular Devices®, USA) using bovine serum albumin (BSA) as standard according to the colorimetric assay^75^.

### Gene expression analysis

Total RNA was extracted with the TRIzol reagent (Life Technologies, CA, USA), following the manufacturer’s instructions, and the obtained samples were treated with an amplification grade DNase I kit (Invitrogen, CA, USA). The High-Capacity cDNA Reverse Transcription kit (Thermo Scientific, Waltham, MA, USA) was used for the complementary DNA synthesis. Quantitative PCR analysis was carried out on the Viia™ 7 Dx PCR-System (Applied Biosystem, CA, USA). The qPCR conditions were 2 min at 50 °C and 95 °C for 10 min; followed by 40 cycles of 95 °C for 15 s and 60 °C for 1 min (annealing temperature of all primers). The mRNA gene expressions were normalized to the *β-actin* and *β-tubulin* using the comparative 2^−∆∆Ct^ method^76^. The primers used are listed in Table 2. In all cases, each qPCR run was performed in triplicate and repeated with at least two independent samples.

**Table 2:** Primer sequences used for qPCR in this study.

Gene	(Sequence 5′–3′)	Eff (%)	References
*β-actin*	F: GCTCCCCCTAGCGTAAATACT	103.22	RNA-Seq by Prado-Lima and Val^42^
	R: TGGACAGGGAGGCCAAGAT		
*β-Tubulin*	F: GACGTGGTGCCCAAAGATGT	100.55	
	R: TGGATGGTGCGCTTGGT		
*Nrf2*	F: CTCCCAATTCAGGCAGATAC	95.38	RNA-Seq by Fé-Gonçalves et al.^79^
	R: CACTGCTTGAACATCCAGG		
*sod1*	F: CAGGACCACACTATAACCCC	102.47	
	R: CTCCCAGCAGTCACATTACC		
*nka α1a*	F: TGCGCCTGAGGATGAGCTTT	95.4	
	R: CAACTGGTGGTTCTGCGCTT		
*Hif-1α*	F: ATCAGCTACCTGCGCATG	97.51	Silva et al.^80^
	R: CTCCATCCTCAGAAAGCAC		

Primers for housekeeping genes (*β-actin* and *β-Tubulin*) and primers for target genes (*Nrf2, SOD1, NKA α1a* and *HIF-1α*).

Eff = Primer efficiency.

### Data analysis

The data are presented as mean ± standard error of mean (SEM; n = 8). Prior to the comparative statistical tests, all the data were examined for normality (Shapiro–Wilk test) and homogeneity of variance (Levene test). Mean differences were evaluated by three-way ANOVA with exposure period, climate scenarios and parasitism as factors, and were discriminated using the Tukey post-hoc test and were considered significant at *p* < 0.05. To better fit the parametric assumptions of ANOVA, data were log-transformed. Data were analyzed by using the Agricolae package in R Software^77^ and graphs were built using ggplot2 package^78^.

## Supplementary Information

Supplementary Legends.

Supplementary Figure 1.
