# Supplementary material for: Climate change affects the parasitism rate and impairs the regulation of genes related to oxidative stress and ionoregulation of Colossoma macropomum
Source: Sci Rep. 2021 Nov 16;11:22350. doi: 10.1038/s41598-021-01830-1 (PMC8595885; doi:10.1038/s41598-021-01830-1)
Supplement: Supplementary file 1 — Supplementary Legends. [file 41598_2021_1830_MOESM1_ESM.doc]

Legend to Supplementary Figure S1

Figure S1. Experimental setup. Juvenile tambaqui were exposed to current and extreme climate room scenario (RCP8.5) and Low (LG) and High (HG) levels of parasitism in each room. After each exposure period, 2 fish were taken from each aquarium in each room (n = 8 fish per treatment). To avoid the volume of water from influencing the rate of parasitism, after removing the fish in 7 days, the volume of water of each aquarium was proportionally reduced. Fish were sampled 7, and 30 days after climate exposure.
