# Supplementary figures and images for: Climate change affects the parasitism rate and impairs the regulation of genes related to oxidative stress and ionoregulation of Colossoma macropomum
Source: Sci Rep. 2021 Nov 16;11:22350. doi: 10.1038/s41598-021-01830-1 (PMC8595885; doi:10.1038/s41598-021-01830-1)

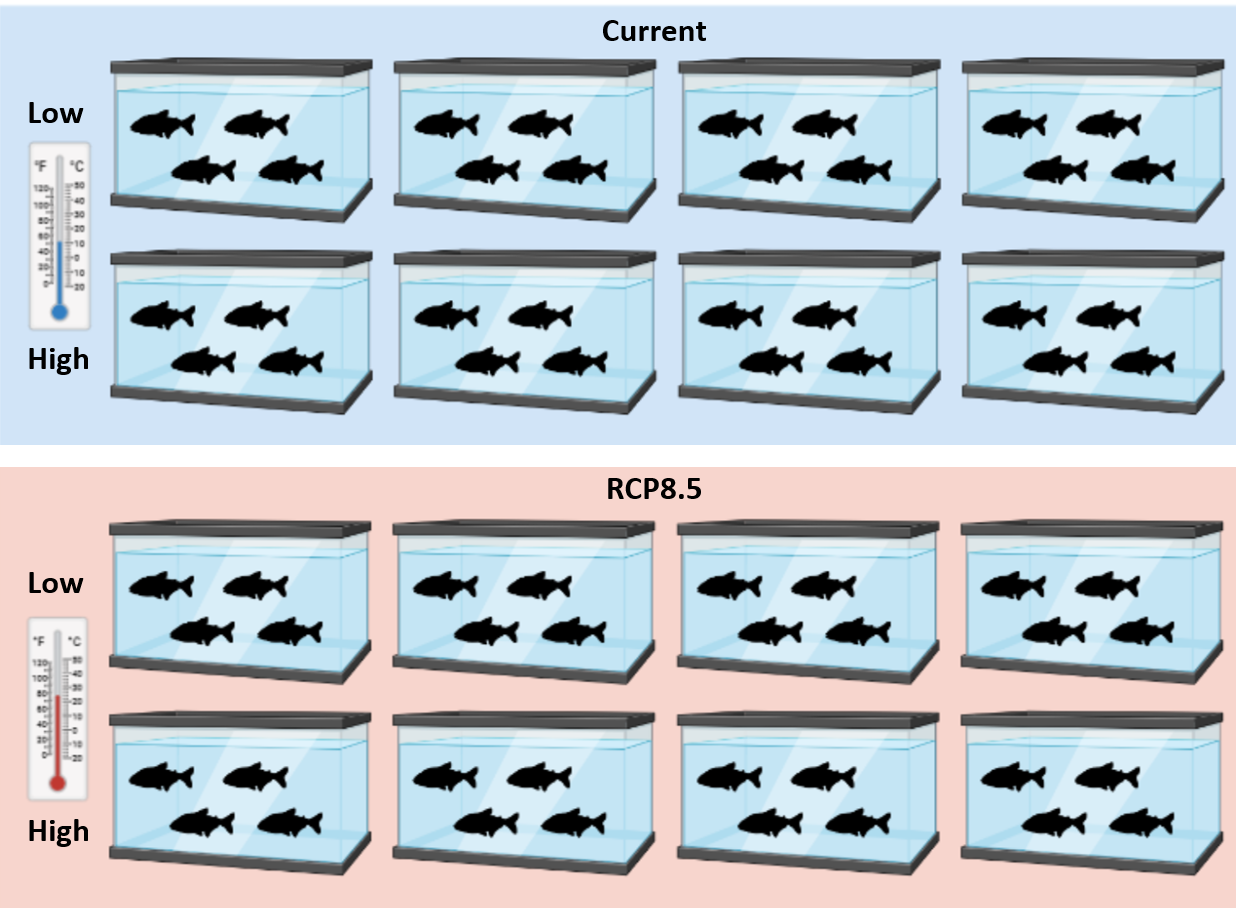

Supplement: Supplementary file 2 — Supplementary Figure 1. [file 41598_2021_1830_MOESM2_ESM.tif]
